# The Photosynthesis and Respiration Efficiency of *Callitriche cophocarpa* Sendtn. Under the Stress of Hexavalent Chromium

**DOI:** 10.3390/ijms27093769

**Published:** 2026-04-23

**Authors:** Barbara Tokarz, Joanna Augustynowicz, Wojciech Makowski, Bartosz J. Płachno, Maksymilian Zienkiewicz, Krzysztof M. Tokarz

**Affiliations:** 1 Department of Botany, Physiology and Plant Protection, Faculty of Biotechnology and Horticulture, University of Agriculture in Krakow, al. Mickiewicza 21, 31-120 Kraków, Poland; joanna.augustynowicz@urk.edu.pl (J.A.); wojciech.makowski@urk.edu.pl (W.M.); 2 Institute of Botany, Faculty of Biology, Jagiellonian University, 9 Gronostajowa St., 30-387 Kraków, Poland; bartosz.plachno@uj.edu.pl; 3 Department of Molecular Plant Physiology, Institute of Environmental Biology, Faculty of Biology, University of Warsaw, I. Miecznikowa 1, 02-096 Warsaw, Poland; mm.zienkiewicz@uw.edu.pl

**Keywords:** aquatic plants, *Callitriche*, chloroplasts, chl *a* fluorescence, dark respiration, hexavalent chromium, photosynthesis, plastids, ultrastructure

## Abstract

The lack of the literature data on the actual CO_2_ assimilation and dissimilation in aquatic plants under conditions of high chromium concentrations prompted this study to determine the efficiency of the photosynthetic apparatus and the actual rates of photosynthesis and respiration in *Callitriche cophocarpa* plants under chromium stress conditions. We hypothesized that *C. cophocarpa* would need to display an efficient acclimation mechanism that allows for efficient carboxylation and dark respiration in the presence of Cr(VI) ions. Shoots of *C. cophocarpa* plants were cultured in the control medium (Cr-free) and in the medium with addition of 0.1 mM potassium chromate. Results revealed that young and mature organs of examined plants respond differently to Cr(VI) ions. In young leaves, the decrease in pigment content (in comparison to control, car, chl *a*, total chl, and chl *b* by 15, 38, 39, and 49%, respectively) and distorted chloroplast ultrastructure led to lower efficiency of photosynthesis (by 22.5% compared to control). These leaves also exhibited reduced dark respiration efficiency (by 36.2% compared to control). In turn, mature leaves exhibited no change in photosynthesis and respiration efficiency. *C. cophocarpa* withstands Cr toxicity due to acclimation strategies associated with the reduction in the size of photosynthetic antennas and the effective use of reduced amounts of incoming radiation, as well as efficient dark respiration in mature leaves.

## 1. Introduction

Globally, about 80% of all industrial wastewater is released into aquatic systems without any pretreatment [1]. Industrial effluents containing metallic elements are a major contributor to water degradation, with an estimated 300–400 Mt of waste discharged into water bodies on an annual basis. Degradation of freshwater resources is considered among the top ten biggest global risks for the past decade [1]. The fundamental process for plants’ functioning is photosynthesis, which is also extremely sensitive to harmful trace metallic ions present in the polluted environment. Aquatic plants, often exposed to metallic pollutants continuously, offer a unique way to study photosynthesis related to metal homeostasis, but there are significantly fewer studies of this phenomenon among aquatic plants compared to terrestrial species.

Chromium occurs in numerous oxidation states, and the most stable forms found in nature are trivalent and hexavalent [2]. Cr(III) exhibits low mobility and easily precipitates and binds to various ligands in the plasma membrane and cell wall. Outside the cell, its toxicity is low [3], whereas Cr(VI) is highly mobile in the wide range of pH, readily crosses biological membranes and, due to its high redox potential, exhibits chemically aggressive nature. This form is extremely dangerous to organisms. Since Cr(VI) is not an essential element, and moreover it is highly toxic, the organisms do not possess transporters dedicated specifically to Cr(VI) uptake. Nevertheless, Cr(VI) is readily transported to plants due to the structural and chemical similarity of its oxyanionic forms to essential nutrient anions: sulfate (SO_4_^2−^) and phosphate (PO_4_^3−^) [4]. Still, molecular mechanisms of Cr ion uptake and transport (structure and distribution of membrane transporters, genetic regulation, etc.) in plants are poorly understood, not to mention those aquatic plants. Once inside a cell, Cr(VI) is easily reduced to Cr(III) and reactive oxygen species formed during this process additionally damage proteins, lipids and nucleic acids [4]. This toxic effect of Cr(VI) in plants leads to delayed seed germination, root damage, reduced biomass, photosynthesis disorders, cell membrane damage, leaf chlorosis, necrosis and ultimately death [5,6].

Submerged aquatic species are particularly susceptible to aquatic pollutants because their entire body surface is permanently exposed to the surrounding water [7,8]. *Callitriche cophocarpa* Sendtn. (water starwort) is a submerged, higher aquatic plant demonstrating an elevated capacity for phytoremediation of Cr(VI)-contaminated water [5]. *C. cophocarpa* is a widespread species found in slow-flowing or stagnant waters in the temperate zones of both hemispheres [9,10,11]. In recent years, our research team has studied the ability of *C. cophocarpa* to bind and detoxify Cr(VI)/Cr(III) [5,12,13,14], and its practical applications in bioremediation [15,16]. The latest study investigated whether the phytoremediation capacity of *C. cophocarpa* plants incubated in Cr(VI) solutions is affected by their symbiotic bacteria [17]. As part of this research, the physiological condition of the plants was examined and certain parameters related to the photosynthetic apparatus were assessed.

When we think about homeostasis and photosynthesis, we consider the internal balance of an organism referring to the autonomous regulation of photosynthesis, which means how a plant maintains good physiological status despite adverse external conditions. Cr(VI) affects photosynthesis in terms of electron transport, photophosphorylation, enzyme activity, and finally CO_2_ fixation [18]. Furthermore, the ions lead to disturbances in the ultrastructure of chloroplasts, such as a poorly developed lamellar system with widely spaced thylakoids and fewer grana, thus disrupting or inhibiting electron transport [6]. In addition, electron transport can also be disrupted by the redirection of electrons from photosystem I (PSI) to Cr(VI) ions [19].

As far as we know there is no available literature containing comprehensive data on the actual rates of carbon dioxide assimilation and dissimilation in aquatic plants under conditions of a high chromium ion concentration. Therefore, the aim of the presented study was to examine the performance of the photosynthetic apparatus and the actual efficiency of photosynthesis and respiration of *C. cophocarpa* young and mature organs under hexavalent chromium stress. We hypothesized that *C. cophocarpa*, as a hyperaccumulator of chromium, would need to display an efficient acclimation mechanism that allows for efficient carboxylation and dark respiration in the presence of Cr(VI) ions.

For the first time, we measured the actual gas exchange in *C. cophocarpa* and examined the ultrastructure of chloroplasts. The results of the study demonstrated that the response of young and mature organs of *C. cophocarpa* to Cr(VI) ion stress, lasting seven days, is distinct. In young leaves, the efficiency of photosynthesis and respiration decreased, while in mature leaves, strategies of acclimatization to the toxic effects of Cr(VI) were associated with reduction in the size of photosynthetic antennas and the efficient use of the reduced amount of radiation reaching the plants.

## 2. Results

### 2.1. Effect of Chromium Stress on Morphology and Biometric Parameters of Callitriche Plants

**Figure 1:**
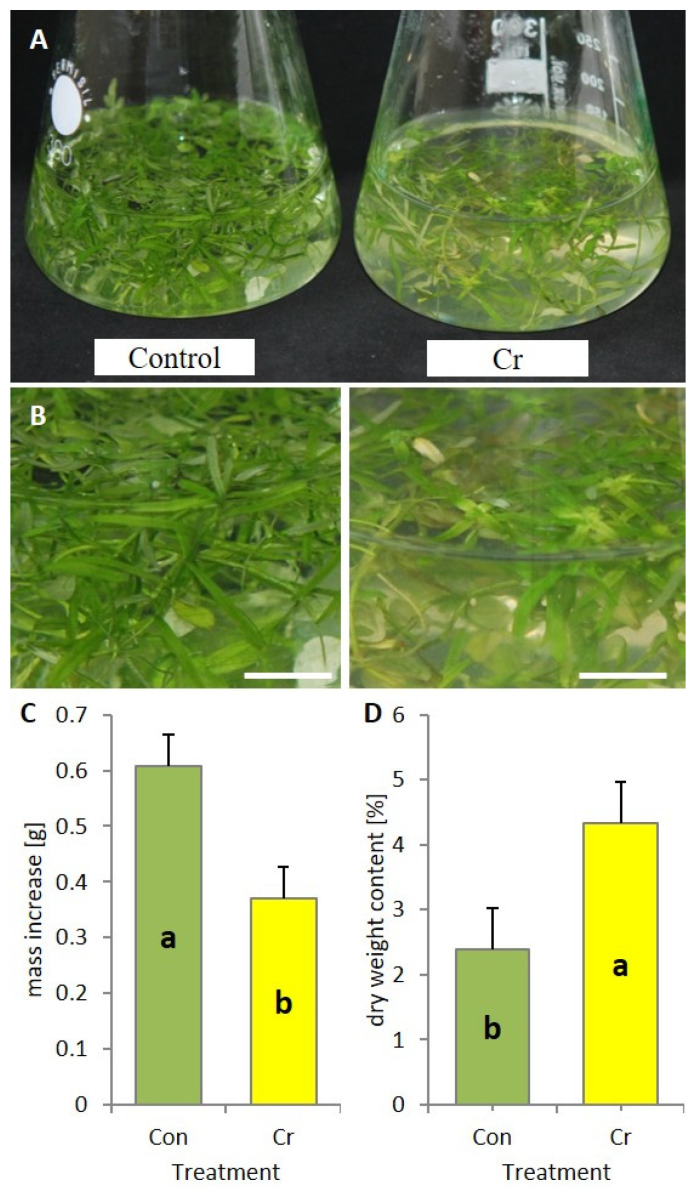
Morphology (**A**,**B**) and biometric parameters (**C**,**D**) of *Callitriche cophocarpa* plants after 7 days of culture in the medium containing Cr(VI) (Con—control, Cr—chromate). (**A**) In vitro culture of *Callitriche* plants; (**B**) close-up on the leaves; (**C**) biomass increase in plants; (**D**) dry weight content of plants (different letters—statistically significant difference at *p* ≤ 0.05, scale bars—1 cm).

Following a seven-day cultivation period, a decline occurred in the growth of *Callitriche* plants cultivated in a medium augmented with chromate (Figure 1A). Plants cultivated on the control medium exhibited green leaves devoid of any indications of impairment (Figure 1B). Conversely, both young and mature leaves of plants cultivated in media containing chromium exhibited a distinct yellowish coloration (Figure 1B). A statistically significant reduction of approximately twofold in the rate of plant biomass increase was observed in media containing chromate, in comparison with the chromium-free medium (Figure 1C). Conversely, the dry weight content of stressed plants exhibited a twofold increase (Figure 1D).

### 2.2. Effect of Chromium Stress on Plasma Membrane Integrity

**Figure 2:**
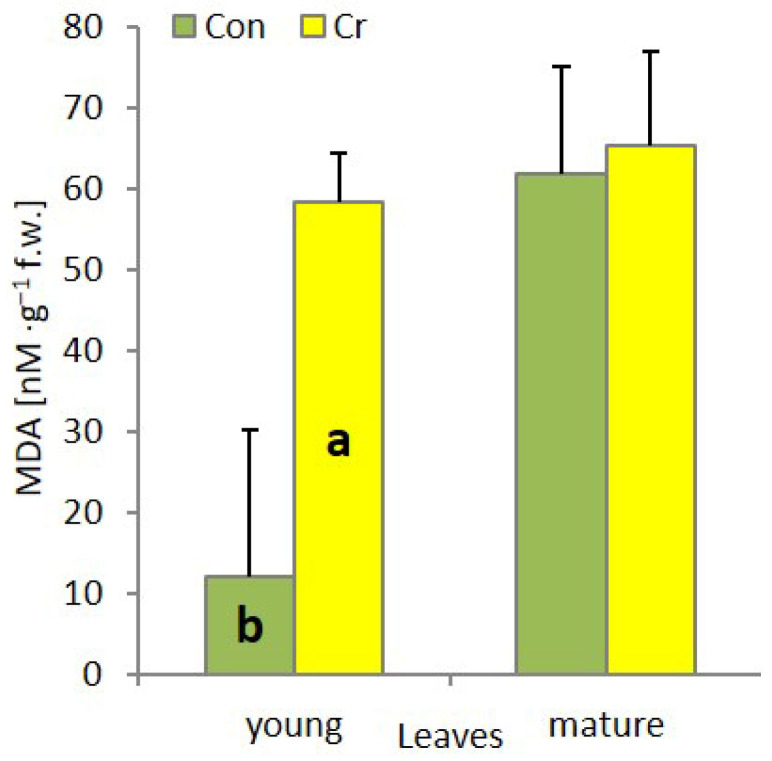
Malondialdehyde (MDA) content in young and mature leaves of *Callitriche cophocarpa* after 7 days of culture in the medium containing Cr (VI) (Con—control, Cr—chromate) (different letters—statistically significant difference within each organ at *p* ≤ 0.05).

An increased MDA (malondialdehyde) content is indicative of elevated lipid peroxidation levels, consequently signaling diminished cell membrane integrity. In the presented studies, the MDA content increased statistically significantly only in the young leaves of plants cultivated in media with the addition of Cr (Figure 2).

### 2.3. Photosynthetic Apparatus Condition Under Chromium Stress

#### 2.3.1. Photosynthetic Pigment Concentration

**Figure 3:**
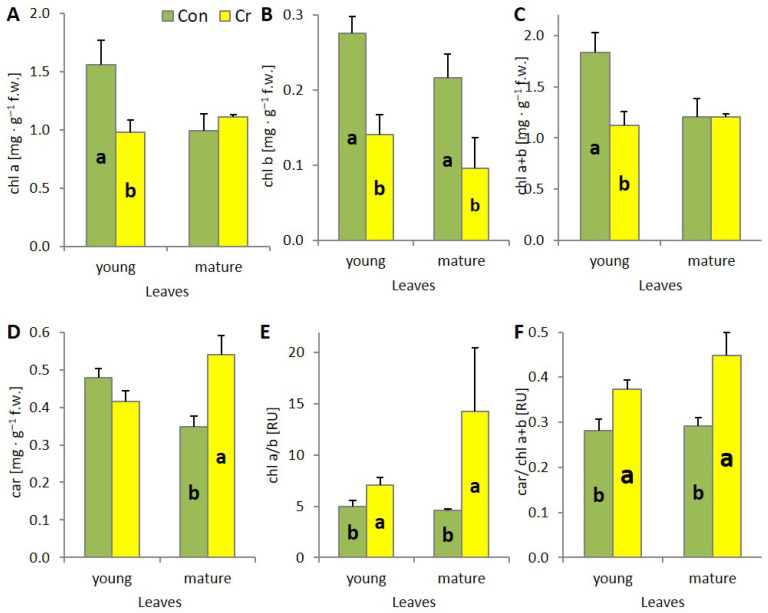
Photosynthetic pigments’ content and photosynthetic pigments’ ratios in young and mature leaves of *Callitriche cophocarpa* after 7 days of culture in the medium containing Cr(VI); (**A**) chl *a* content; (**B**) chl *b* content; (**C**) chl *a* + *b* content; (**D**) car content; (**E**) chl *a*/*b* ratio; (**F**) car/chl *a* + *b* ratio (Con—control, Cr—chromate) (different letters—statistically significant difference within each organs at *p* ≤ 0.05, RU—relative units).

A significant decrease in photosynthetic pigment content was observed in young leaves of *Callitriche* plants cultivated in media including Cr(VI). The total chlorophyll content and chlorophyll *a* and chlorophyll *b* content in leaves on Cr-containing media decreased approximately twofold compared to control leaves (Figure 3A–C). No change in carotenoid content was observed in the young leaves of plants grown in media supplemented with chromium. Consequently, changes in chlorophyll pigment content led to a threefold increase in the chl *a*/*b* ratio and an increase in the car/chl *a* + *b* ratio in young leaves of plants incubated in Cr medium (Figure 3E,F). In contrast, mature leaves of plants grown in Cr-containing media showed a twofold decrease in chlorophyll *b* content and a threefold increase in the chl *a*/*b* ratio (Figure 3B,E). A significant increase in the carotenoid content in the leaves of plants cultivated in Cr medium and an increase in the car/chl *a* + *b* ratio were also observed (Figure 3D,F).

#### 2.3.2. Chl *a* Fluorescence Parameters

**Figure 4:**
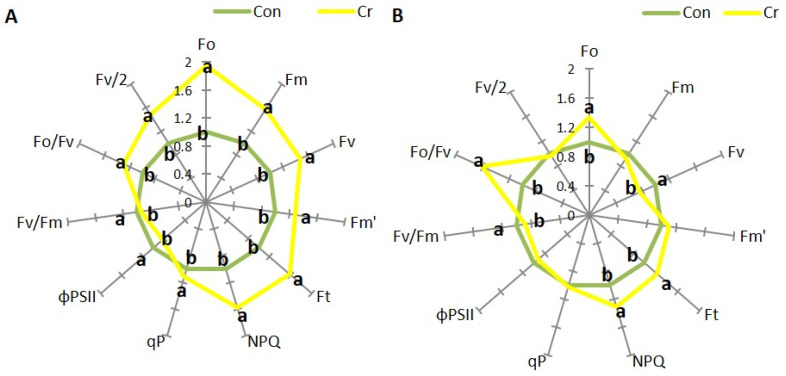
Chlorophyll *a* fluorescence parameters of young (**A**) and mature (**B**) leaves of *Callitriche cophocarpa* after 7 days of culture in the medium containing Cr (VI) (Con—control, Cr—chromate) (all the values were expressed relative to the control (set as 1); different letters—statistically significant difference at *p* ≤ 0.05).

The presence of chromium in the medium caused a significant increase in both basic (Fo), maximum (Fm, Fm′), variable (Fv) and transient (Ft) fluorescence in young leaves compared to control (Figure 4A, Table S1). Non-photochemical fluorescence quenching (NPQ), photochemical fluorescence quenching (qP), plastoquinone pool (Fv/2) and efficiency of the water-splitting complex of photosystem II (PSII) (Fo/Fv) also increased in these leaves, while the efficiency of PS II photochemistry (ΦPSII) and maximum quantum field of PSII (Fv/Fm) decreased (Figure 4A, Table S1). In contrast, in mature leaves of Cr-treated plants, basic (Fo) and transient (Ft) fluorescence increased but variable (Fv) fluorescence decreased (Figure 4B, Table S1). In mature leaves, a decrease in Fv/Fm and an increase in Fo/Fv were observed. In these leaves, however, an increase in NPQ and no change in ΦPSII and Fv/2 were recorded (Figure 4B, Table S1).

#### 2.3.3. Gas Exchange

**Figure 5:**
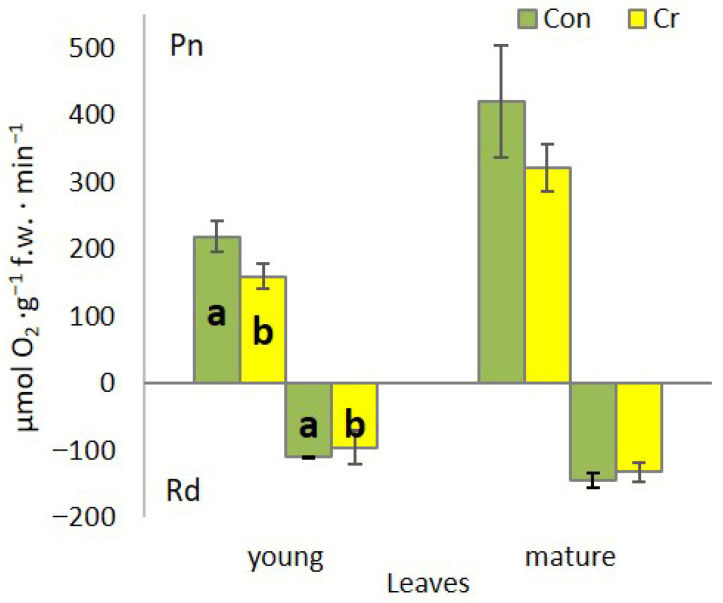
Gas exchange of *Callitriche cophocarpa* young and mature leaves after 7 days of culture in the medium containing Cr(VI) (Con—control, Cr—chromate); Pn—rate of net photosynthetic efficiency as O_2_ release; Rd—rate of dark respiration efficiency as O_2_ uptake (different letters—statistically significant difference within each organ and parameters at *p* ≤ 0.05).

Measurements of O_2_ release and uptake by *Callitriche* plants demonstrated significant differences between plants cultivated in media with and without chromium addition. Apical shoot fragments with young leaves exhibited a decrease in the photosynthesis rate (O_2_ release) and respiration rate (O_2_ uptake) (Figure 5). However, in shoot fragments with mature leaves, photosynthesis and respiration did not change significantly in the medium with Cr in comparison to control (Figure 5).

#### 2.3.4. Chloroplast Ultrastructure

**Figure 6:**
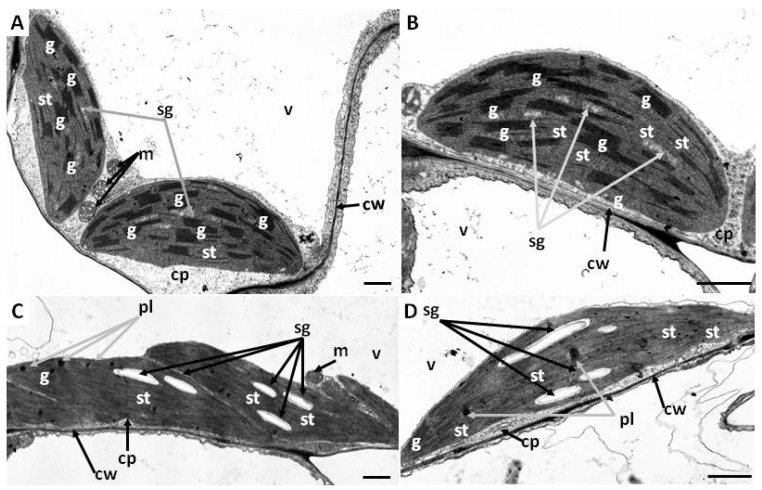
Chrome (Cr) effects on the chloroplast ultrastructure of *Callitriche cophocarpa* young leaves. (**A**,**B**) chloroplasts of plants from control medium; (**C**,**D**) chloroplasts of plants from Cr medium. Abbreviations: cp—cytoplasm, cw—cell wall, g—granum, m—mitochondrion, pl—plastoglobule, sg—starch grain, st—stroma thylakoid, v—vacuole; scale bars: 1 μm.

**Figure 7:**
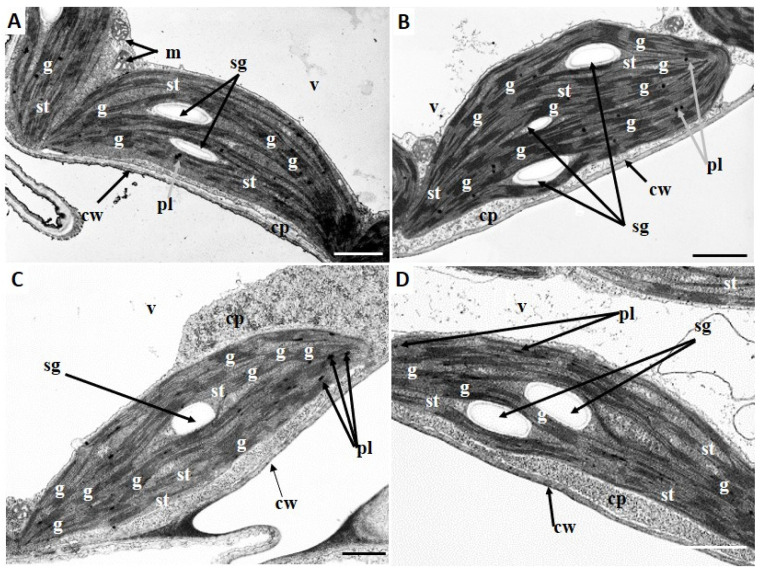
Chrome (Cr) type effects on the chloroplast ultra-structure of *Callitriche cophocarpa* mature leaves. (**A**,**B**) chloroplasts of plants from control medium; (**C**,**D**) chloroplasts of plants from Cr medium. Abbreviations: cp—cytoplasm, cw—cell wall, g—granum, m—mitochondrion, pl—plastoglobule; sg—starch grain, st—stroma thylakoid, v—vacuole; scale bars: 1 μm.

Chloroplasts in both young (Figure 6A,B) and mature (Figure 7A,B) leaves of *Callitriche* plants from control conditions were lenticular in shape. Chloroplasts from these leaves exhibited a rich, well-organized system of internal membranes with numerous, well-developed grana (Figure 6A,B and Figure 7A,B). However, chloroplasts from mature leaves exhibited a higher proportion of stromal thylakoids, along with numerous starch grains and some plastoglobules (Figure 7A,B). In chloroplasts from young leaves, plastoglobules were absent and significantly fewer formulating starch grains were observed (Figure 6A,B). Chloroplasts in young leaves treated with chromate exhibited well-visible starch grains and some large plastoglobules (Figure 6C,D). Additionally, the internal membrane system was severely damaged, exhibiting few, barely visible grana and numerous stroma thylakoids (Figure 6C,D). Moreover, chloroplasts in leaves from media with chromium showed a slightly altered, flattened and elongated shape (Figure 6C,D). In chloroplasts of mature leaves from media supplemented with chromium, large starch grains were observed (Figure 7C,D). Furthermore, in contrast to young leaves, chloroplasts of mature leaves from Cr medium had no damaged internal membrane systems and rarely visible plastoglobules (Figure 7C,D).

## 3. Discussion

The maximum permissible Cr(VI) content in surface water is 0.02 mg/L according to the legal requirements Journal of Laws, regulation of the Ministry of Infrastructure of Poland from 21 June 2021 [20]. The recommended safe levels of hexavalent chromium concentrations for freshwater and marine organisms are 0.001 mg/L, and for water used for irrigation they are 0.008 mg/L [21]. However, in polluted areas, mainly in river waters where wastewater from the metallurgical, electroplating and engraving industries is discharged, the concentration of Cr(VI) is several thousand times higher [2,21,22]. Aquatic plants play an important role in the uptake, storage and recycling of metallic elements. Furthermore, the ability of plants to accumulate chromium within themselves is considered one of the most promising methods for the remediation of chromium-contaminated areas [23]. Unlike animals and humans, chromium is not an essential element for plants [24,25].

One of the most visible symptoms of the toxic effects of chromium on plants is the retardation or inhibition of plant growth and development, manifested by a decrease in biomass [26]. The results of the presented studies also showed a significant reduction in biomass growth of plants grown in media with chromate addition (Figure 1C). Disorders in the growth of plant organs—in the case of *Callitriche*, mainly stems and leaves—result from various harmful effects of chromium ions [25,26,27]. Firstly, due to their variable valence, chromium ions interact with essential nutrients (such as zinc, iron, calcium, magnesium, manganese and copper) and interfere with their uptake, thus affecting the mineral nutrition of plants [25,28]. Secondly, chromium disrupts the signaling pathways of hormones involved in cell division and elongation, as well as the functioning of enzymes such as cellulase synthase and lignin biosynthesis enzymes, which are essential for stem development [26,27]. Furthermore, once chromium ions enter cells, they generate reactive oxygen species (ROS), leading to damage to proteins, lipids and DNA, which may result in cell apoptosis [26]. Interestingly, our results showed a statistically significant increase in the percentage of dry matter in *Callitriche* shoots growing under Cr(VI) stress conditions (Figure 1D). In general, heavy metals, including chromium, cause photosynthesis disorders, leading to a decrease in dry matter content, 80–90% of which consists of carbon compounds produced by plants [25]. In plants resistant to the toxic effects of heavy metals, the products of photosynthesis are utilized in pathways involved in the synthesis of secondary metabolites. These include phenolic derivatives that are incorporated into the cell wall and permanently bind heavy metals, while simultaneously increasing the plant’s dry matter content [29,30].

Photosynthesis and respiration represent two fundamental metabolic processes, the disruption of which can lead to plant death. The toxic effect of Cr(VI) on these processes is associated with the direct toxic effect of this oxyanion on the synthesis pathway and the induction of the degradation of photosynthetic pigments, damage to the PSII and PSI reaction centers (RC), or, common to both processes, damage to the structure and function of plasma membranes and cytochrome systems. Consequently, there is a substantial decline in the efficiency of CO_2_ assimilation and the intensity of mitochondrial respiration [27]. In *C. cophocarpa* young leaves, Cr(VI) caused a significant decrease in the content of chl *a*, chl *b* and total chlorophylls, with no change in carotenoid content. The decrease in chl *a* and *b* content triggered changes in the ultrastructure of chloroplasts. Chlorophyll pigments, together with docking proteins, are a key element responsible for the structure and function of the photosynthetic apparatus: 70–80% of chlorophylls are localized in the LHC-PSII supercomplex [31]. The presence of this complex, apart from utilizing solar radiation and generating a proton gradient, is responsible for the formation and ultrastructural functionality of grana thylakoids [32]. The presence of Cr (VI), leading to a decrease in chlorophyll *a* and *b* content, caused a structural and functional reorganization of chloroplasts, where the number of grana thylakoids decreased and the number of stroma thylakoids increased (Figure 6C,D). As a result of the above-mentioned changes, both the number of active PSII reaction centers (Fo) and the size of LHCII (Light harvesting complex of PSII) antennae decreased. Aforementioned changes resulted either from direct substitution of Mg^2+^ ions in the porphyrin ring of chlorophyll, and/or direct toxic effects of Cr (VI) on protochlorophyllide reductase and aminolevulinic acid dehydratase—key enzymes in the chlorophyll synthesis pathway [33,34]. At the same time, in the photosynthetic apparatus of young leaves, an increase in the efficiency of OEC function and an increase in the efficiency of the rapidly reducing pool of plastoquinone (Fv/2) were observed. Observed functional changes indicate, on the one hand, the absence of limitation in electron transport on the donor side of PSII as well as the high potential to effectively discharge limitation on its acceptor side [35]. We observed an increase in the activity of the fast-reducing PQ (plastoquinone) pool, resulting from a rapid decrease in the number of active PSII RC with an unaffected number (10–15) of active PQ molecules per single PSII RC [36]. However, it seems that, as in other studied species, the toxic effect of Cr (VI) also manifests itself by very effective blocking of linear electron transport at the level of Q_A_, Q_B_ and cyt_b6f_ in young leaves of *Callitriche* [27]. As a result, despite the increase in the efficiency of dissipation of excess absorbed energy (increase in NPQ) in young leaves, redox homeostasis was exceeded and thylakoid membranes were degraded, as evidenced by the increase in MDA and large plastoglobules in the ultrastructure of chloroplasts (Figure 6C,D). Cr(VI), in addition to disrupting the photosynthetic apparatus, causes structural and functional changes in the mitochondria. Cr ions induce damage to the outer mitochondrial membrane, crystallization of the mitochondrial matrix, and interact directly with cytochromes. As a result, a decrease in the functioning of the electron transport chain is observed [37]. All these changes induced by the presence of Cr(VI) led to a significant decrease in the efficiency of photosynthesis as well as mitochondrial respiration (Figure 5).

In mature leaves, the influence of Cr(VI) caused a significant decrease in chl *b* content with no change in chl *a* levels. Chl *b* is a key component of photosynthetic antennae, and a decrease in its content indicates a decrease in their size and consequently a reduction in the efficiency of radiation absorption [38]. Chl *a* is included, together with chl *b*, in the composition of antennae, but the most significant role of chl *a* is related to its presence in PSII RC and PSI RC where it determines the possibility of photochemical conversion of absorbed radiation [39]. No change in chl *a* content indicates effective protection, primarily of the chlorophyll synthesis pathway as well as the photosynthetic apparatus itself, from the toxic effects of Cr(VI). However, the protection efficiency of PSII RC itself is not absolute, as evidenced by a significant increase in inactive PSII RC (Fo). However, the efficiency of RC is not affected (qP, ΦPSII), due to significantly higher OEC efficiency (Fo/Fv), as well as electron transport outside PSII RC [16], which protects PSII from photooxidation on both the donor and acceptor sides. Higher electron supply from OEC, together with the unchanged size of the fast-reducing PQ pool (Fv/2), can lead to overloading of linear electron transport and photooxidation of PSII RC [38]. Previous studies on *Callitriche* demonstrated that, in mature Cr(VI)-treated plants, there was no disruption of electron transport around PSI [17]. Increased carotenoid content, primarily associated with the xanthophyll cycle, is associated with efficient dissipation of excess converted energy in a thermal manner (NPQ). As a result, mature organs exhibited efficient photosynthesis, as well as dark respiration.

Cr content in young and mature leaves of *C. cophocarpa* may reach the level of c.a. 500–600 mg·kg^−1^ d.w. and differ slightly between organs [5,40]. Higher Cr levels (up to 10%) may be accumulated in younger leaves due to their greater capacity to reduce Cr(VI) to Cr(III) [40]. Once reduced to Cr(III), chromium is more readily bound, as a greater number of functional groups are available for binding at this oxidation state [40]. Consequently, the differences in photosynthetic and mitochondrial respiratory rates observed in this study may result from greater chromium accumulation in young leaves. Our earlier studies demonstrated that Cr could affect the levels of Ca, Cl, K, and S in leaves [14,28]. However, they also revealed that Cr had no effect on the Mg concentration (a component of chlorophyll) in the stomatal apparatus [14]. Cr exhibits strong redox activity and, in the form of Cr(VI), is highly toxic to plants (and other organisms); however, its complex electronic chemistry has been a major obstacle in elucidating its toxicity mechanisms. Therefore, research using *Callitriche* sp. as model organisms in aquatic plants to study Cr(VI) homeostasis at the genetic, metabolic, and ionic levels is continued.

## 4. Materials and Methods

### 4.1. Plant Material

Experimental material comprised in vitro cultures of *Callitriche cophocarpa* Sendtn. (water-starwort). Cultures were initiated from shoot fragments of plants collected from Dłubnia River, in Southern Poland (50°16′ N 19°56′ E). Plants were cultivated according to the protocol established by Piwowarczyk and Hanus-Fajerska [41] in vessels containing 150 mL of liquid MS medium [42] with 10 g L^−1^ sucrose and pH 5.8, under a 16/8 h (day/night) photoperiod, light intensity of 70 μmol × m^−2^ × s^−1^ photosynthetic photon flux density (PPFD), and 21 ± 1 °C temperature.

### 4.2. Chromium Stress Treatments

Hexavalent chromium in the form of potassium chromate (K_2_CrO_4_) (Cr) at a concentration of 0.1 mM of Cr was added to the culture medium prior to autoclaving; then, pH equal to 5.8 was established. The applied Cr concentration was selected based on previous studies [5], which showed that this concentration causes stress but does not kill *C. cophocarpa* plants. Control plants were cultured in chromium-free medium (Con). The light and temperature conditions were as described above. For each treatment, approximately 1 g of fresh weight of plant shoots was cultivated in each of vessels. Due to the full control of environmental conditions, a completely randomized design of experiment was used.

### 4.3. Chromium Stress Assessment

After 7 days of culture, the chromium impact on *Callitriche* plants was evaluated. Plant biomass from each vessel was weighed and mass increment was calculated. Parts of fresh shoots were weighed directly after collection, dried for 48 h in 120 °C and reweighed. The percentage content of dry weight in the plant tissue was calculated.

Young and mature leaves were collected from plants in each treatment. Young leaves were collected from the first and second node under the apical bud. Mature leaves were collected from the fifth, sixth and seventh node under the apical bud.

### 4.4. Lipid Peroxidation Estimation

The level of lipid peroxidation in leaves was evaluated based on malonylodialdehyde (MDA) concentration. MDA concentrations were determined spectrophotometrically according to Dhindsa et al. [43]. Approximately 10 mg of collected leaves were homogenized in 0.5 mL of 0.1% trichloroacetic acid (TCA). After centrifugation (4 °C, 5 min, 10,000 g), supernatant (0.2 mL) was mixed with 0.8 mL of 20% TCA with 0.5% of TBA (thiobarbituric acid), incubated at 95 °C (30 min), immediately cooled on ice, and centrifuged (4 °C, 10 min, 10,000 g). The absorbance of the supernatant was measured at 532 nm and 600 nm. The value at 532 nm (A_532_) was reduced by the value at 600 nm (A_600_, the correction value): A_x_ = A_532_ − A_600_. The concentration of MDA was calculated using the absorbance coefficient for MDA ε = 155 mM^−1^cm^−1^.

### 4.5. Photosynthetic Pigment Content Estimation

Photosynthetic pigment content was determined in collected leaves according to spectrophotometric methods of Lichtenthaler [44]. Fresh tissue samples (approximately 10 mg) were homogenized in 1 mL of 80% acetone under ice conditions. After centrifugation (15 min, 4800 g, 4 °C), the absorbance of the extract was measured at 470, 646 and 663 nm to determine chlorophyll *a* (chl *a*), chlorophyll *b* (chl *b*) and total carotenoid (car) content, respectively. The pigment content was calculated according to Wellburn [45]. Additionally, total chlorophylls (chl *a* + *b*), the chlorophyll a/b ratio (chl *a*/chl *b*), and the ratio of total carotenoids to total chlorophylls (car/chl *a* + *b*) were also calculated.

### 4.6. Chlorophyll a Fluorescence Measurements

The chlorophyll *a* fluorescence induction curve analysis was performed using a chlorophyll fluorescence-monitoring system (FluorCam; Photon Systems Instruments, Drásov, Czech Republic). Ten young and ten mature leaves were dark-adapted for 20 min, and fluorescence was induced by applying a 0.8 s saturating red light pulse (2000 µmol m^−2^ s^−1^). Selected chlorophyll fluorescence parameters were measured and calculated (Table 1).

### 4.7. Measurements of O_2_ Exchange

Control and hexavalent chrome (Cr(VI))-treated shoot fragments (bearing young or mature leaves) were kept in darkness (for approximately 30 min) in the water. Then, apical shoot fragments were transferred into an O_2_ electrode chamber (10—mL volume) with a water Clark-type electrode (TriOxmatic EO 200; WTW GmbH, Weilheim, Germany) and dark respiration (R0) before photosynthesis (Pn) was recorded. Afterward, 100 uL of pure CO_2_ was injected into the chamber (to provide the necessary substrate for the dark phase of photosynthesis), and the apical fragments were exposed to white light (60 umol photons m^−2^ s^−1^) and O_2_ evolution was monitored. O_2_ evolution and uptakes were monitored during the light and dark cycles (usually three to four cycles). The temperature inside the chamber was 25 ± 1 °C during the dark and light periods. The respiratory O_2_ uptake (Rn) after Pn was determined after approximately 1 min of darkness. The rates of O_2_ evolution and uptake were expressed in terms of fresh weight.

### 4.8. TEM Observation

After 7 days of culture, young and mature leaves were fixed in 2.5% (*v*/*v*) glutaraldehyde/4% (*v*/*v*) formaldehyde in 0.1 M sodium cacodylate buffer (pH 7.0) for 2 h at 4 °C, washed three times in 0.1 M sodium cacodylate buffer and post-fixed in 1.5% (*w*/*v*) osmium tetroxide solution for 1.5 h at 0 °C. This was followed by dehydration using a series of graded ethanol solutions, infiltration, embedding using an epoxy embedding medium kit (Honeywell Fluka, Charlotte, NC, USA), and polymerization at 60 °C. Next, 70 nm thick sections were cut using a Leica ultracut UCT ultramicrotome (Leica Camera, Weztlar, Germany), stained with uranyl acetate and lead citrate [46]. These ultrathin sections were examined using a transmission electron microscope Hitachi H500 (Hitachi, Tokyo, Japan) at an accelerating voltage of 75 kV.

### 4.9. Statistical Analyses

Plants for each treatment (control and Cr) were cultivated in four vessels and the experiment was repeated twice. Biometrical parameters were calculated from three to five replicates. All the spectrophotometric estimations (photosynthetic pigments and MDA) were made in three to five replications. Gas exchange measurements were made in three to five and chl *a* fluorescence measurements were made in seven to fifteen replications. Significant differences between means were indicated using Student’s *t*-test at *p* < 0.05. STATISTICA 13.3 (StatSoft Inc., Tulsa, OK, USA) was used to carry out statistical analyses.

## 5. Conclusions

Presented result revealed that young and mature organs of *Callitriche cophocarpa* respond differently to Cr(VI) ion stress lasting 7 days. In young leaves, photosynthetic efficiency decreases, which results from a reduction in pigment content leading to changes in the ultrastructure of chloroplasts and impairment of the photosynthetic apparatus. The decrease in respiration efficiency also indicates Cr(VI)-induced damage to mitochondria in these leaves. In turn, changes in the amount of chl *b* and carotenoids, as well as chl *a* fluorescence parameters, indicate that the acclimation strategies of mature organs to the toxic effects of Cr(VI) are associated with reduction in the size of photosynthetic antennas, the effective use of reduced amounts of incoming radiation. Furthermore, the lack of limitation on the donor (Fo/Fv) and acceptor side (qP, ΦPSII) of PSII leads to no damage to the structure and function of the photosynthetic apparatus (no changes in MDA) and the maintenance of efficient photosynthesis (Pn). Efficient photosynthesis is accompanied by equally efficient dark respiration.

It is important to consider that in the plant, the process of photosynthesis is also carried out by non-leaf organs (stems), which may have a significant advantage over leaves under stress conditions. This is due to their structure (reduced surface area with increased volume), which allows for more efficient sequestration of harmful ions in the cell walls of non-photosynthesizing cells. Efficient stem photosynthesis enables the plant to generate the energy needed to distribute harmful ions, as well as providing assimilates for further growth.

## Figures and Tables

**Table 1:** Abbreviations and descriptions of extracted and calculated fluorescence parameters.

Parameter	Definition
Fo	basic chlorophyll fluorescence yield after dark adaptation
Fm	maximal chlorophyll fluorescence yield at saturating light pulse
Fv	variable fluorescence, Fv = Fm − Fo
Fm′	maximum fluorescence yield during illumination with a saturating pulse during actinic light
Ft	level of fluorescence during illumination
qP	photochemical fluorescence quenching, qP = (Fm′ − Ft)/(Fm′ − Fo)
NPQ	nonphotochemical fluorescence quenching, NPQ = (Fm − Fm′)/Fm′
ΦPSII	effective photochemical quantum yield of PSII, ΦPSII = (Fm′ − Ft)/Fm′

## Data Availability

The raw data supporting the conclusions of this article will be made available by the authors upon request.

## Supplementary Materials

The following supporting information can be downloaded at https://www.mdpi.com/article/10.3390/ijms27093769/s1.

## Abbreviations

LHC-PSII/LHCII	Light harvesting complex of PSII
MDA	Malondialdehyde
OEC	Oxygen evolving complex
PSI	Photosystem I
PSII	Photosystem II
PSI RC	PSI reaction center
PSII RC	PSII reaction center
PQ	plastoquinone
