# Supplementary material for: The Photosynthesis and Respiration Efficiency of Callitriche cophocarpa Sendtn. Under the Stress of Hexavalent Chromium
Source: Int J Mol Sci. 2026 Apr 23;27(9):3769. doi: 10.3390/ijms27093769 (PMC13164116; doi:10.3390/ijms27093769)
Supplement: Supplementary file 1 [file ijms-27-03769-s001.zip › ijms-4200306-supplementary.pdf]

Table S1. Raw values of chlorophyll *a* fluorescence parameters of young (A) and mature (B) leaves of *Callitriche cophocarpa* after 7 days of culture in the medium containing Cr (VI) (Con – control, Cr – chromate) **different letters** - statistically significant difference at $p \leq 0.05$ within each organs).

Parameter [RU]	Young Leaves		Mature Leaves	
	Con	Cr	Con	Cr
Fo	145 ± 20 **b**	283 ± 31 **a**	261 ± 60 **b**	351 ± 47 **a**
Fm	718 ± 90 **b**	1129 ± 81 **a**	1058 ± 199	960 ± 189
Fv	573 ± 85 **b**	846 ± 71 **a**	789 ± 140 **a**	659 ± 153 **b**
Fm′	441 ± 57 **b**	567 ± 43 **a**	565 ± 137	609 ± 148
Ft	191 ± 21 **b**	302 ± 20 **a**	258 ± 58 **b**	332 ± 57 **a**
NPQ	0.63 ± 0.06 **b**	1.00 ± 0.10 **a**	0.90 ± 0.17 **b**	0.94 ± 0.45 **a**
qP	0.85 ± 0.02 **b**	0.95 ± 0.08 **a**	1.01 ± 0.05	0.93 ± 0.24
φPSII	0.57 ± 0.03 **a**	0.47 ± 0.04 **b**	0.54 ± 0.02	0.45 ± 0.07
Fv/Fm	0.80 ± 0.03 **a**	0.75 ± 0.02 **b**	0.75 ± 0.04 **a**	0.63 ± 0.08 **b**
Fo/Fv	0.26 ± 0.06 **b**	0.34 ± 0.04 **a**	0.33 ± 0.04 **b**	0.61 ± 0.21 **a**
Fv/2	286 ± 42 **b**	423 ± 35 **a**	395 ± 70	361 ± 84
